# 교대 근무 형태에 따른 간호사의 수면의 질, 불면증, 만성피로 및 업무 성과: 횡단적 비교연구

**DOI:** 10.7586/jkbns.26.015

**Published:** 2026-08-31

**Authors:** Ji Eun Lee, Dong Yeon Kim, Hye Hyeon Shin, Su Hyeon Kim, Hee Young Choi

**Affiliations:** Nursing Department, The Catholic University of Korea, Seoul St. Mary's Hospital, Seoul, Korea; 가톨릭대학교 서울성모병원 간호부

**Keywords:** Fatigue, Shift work schedule, Sleep quality, Sleep wake disorders, Work performance, 피로, 교대근무, 수면의 질, 불면증, 업무수행

## Abstract

**Purpose:**

This study compared sleep quality, insomnia, chronic fatigue, and work performance between nurses working fixed night shifts and those working rotating three-shift schedules and examined the relationships among these variables by work schedule type. Factors associated with work performance were also identified.

**Methods:**

This cross-sectional survey included nurses working at a university hospital. Data were collected using structured questionnaires measuring sleep quality, assessed with the Korean version of the Pittsburgh Sleep Quality Index, insomnia, chronic fatigue, and work performance. Data were analyzed using descriptive statistics, nonparametric tests, Spearman’s correlation coefficients, and hierarchical multiple regression analysis using SPSS version 30.0 program.

**Results:**

Insomnia differed significantly between nurses working fixed night shifts and those working rotating three-shift schedules. Among nurses on rotating three-shift schedules, poorer sleep quality, greater insomnia, and higher chronic fatigue were significantly associated with lower work performance, whereas sleep-related variables were not significantly associated with work performance among fixed-night-shift nurses. Hierarchical multiple regression analysis showed that education level, job satisfaction and fixed-night-shift work were significant factors associated with work performance. The final model explained 17.0% of the variance in work performance, with an adjusted R^2^ of .15. Job satisfaction showed the strongest association.

**Conclusion:**

The relationship between sleep-related factors and work performance differed by work schedule type. These findings suggest that interventions to improve nurses’ work performance should combine sleep health management with organizational strategies, particularly efforts to improve job satisfaction. The role of fixed-night-shift nursing systems should be examined further in future studies.

## 서론

### 1. 연구의 필요성

교대근무는 간호사의 직무 특성상 불가피한 근무 형태이며, 선행연구에 따르면 특히 야간근무는 인간의 일주기 리듬(circadian rhythm)을 교란시키는 주요 환경 요인으로 보고되고 있다[1]. 야간근무로 인한 수면 시점의 변화와 불규칙한 근무패턴은 수면의 질 저하, 수면 잠복기 증가, 수면 효율 감소 및 주간 기능 저하 등 다양한 수면 문제와 관련될 수 있다[2]. 반복적인 야간근무는 수면-각성 주기의 불규칙성과 수면 건강 저하와 관련될 수 있으며, 이는 피로, 주의력 저하 및 업무수행 부담으로 이어져 수면의 질 저하, 수면 잠복기 증가, 수면 효율 감소 등 다양한 수면 문제가 발생하는 것으로 보고되고 있다[3].

체계적 문헌고찰 연구에 따르면 미혼의 젊은 성인이 야간근무에 오랫동안 노출될수록, 야간근무 후 휴무가 짧을수록, 20년 이상 야간근무를 지속할수록 유방암과 조기폐경 등의 질환 발병률이 높아지는 것으로 나타났다[4]. 야간근무를 하는 간호사들은 규칙적인 수면이 불가능하여 잠들기 어려움, 수면 지속의 어려움, 수면 후 회복의 어려움, 수면에 대한 만족도 저하, 일어나기 어려움 등 전반적인 수면의 질 저하를 경험한다[5,6].

수면은 에너지 항상성 회복과 신경인지 기능 유지에 필수적인 생리적 과정이며, 수면 효율 감소와 수면 잠복기 증가는 불면증의 주요 특성으로 간주된다. 특히, 수면시간 6시간 미만, 수면 잠복기 30분 이상, 수면 효율 85% 미만은 불면증의 객관적 지표로 제시된다[3]. 이러한 불면증은 인지기능 저하, 반응시간 지연, 집중력 감소를 초래하며, 수면 후에도 피로가 회복되지 않는 상태를 유발한다[7]. 나아가 지속된 불면증은 만성피로로 이어져 판단력 저하와 주의력 감소를 초래하며, 이는 직무 수행 능력 저하로 연결될 수 있다[5]. 또한, 전반적으로 기운이 없어 일상적인 활동을 수행할 수 없는 상태인[5] 만성피로도 인지기능 저하와 집중력 감소를 초래하여 직무 수행 능력에 부정적인 영향을 미친다. 특히 간호사의 경우 이러한 변화는 투약 오류, 의료기기 조작 오류, 주사침 손상 등 환자 및 간호사 안전과 직결되는 문제로 이어질 수 있다[8].

최근 야간전담근무 제도가 확대 시행되면서, 고정된 야간근무 형태가 간호사의 수면 건강, 피로 및 업무성과와 어떠한 관련성을 보이는지 규명할 필요가 있다. 야간전담근무는 일정 기간 동일한 시간대에 근무한다는 점에서 3교대근무와 구별되지만, 야간근무 노출이 지속된다는 점에서 수면 문제와 피로의 부담도 함께 고려할 필요가 있다[9].

기존 연구에서는 수면의 질 저하가 업무성과 감소와 관련된 것으로 보고되었으나[10], 고정된 전담근무 형태는 업무의 연속성과 일관성을 유지할 수 있고, 특정 근무 환경에 대한 적응과 숙련도를 향상시킬 수 있다는 보고도 있다[11]. 특히 야간전담근무는 상대적으로 업무량이 감소하고 근무 환경이 안정적인 특성을 가질 수 있으며, 이러한 근무 여건은 직무 집중도를 높여 간호업무의 효율성과 성과 향상에 긍정적인 영향을 미칠 가능성도 있다[10]. 최근 연구에서도 야간근무는 수면 문제와 관련이 있지만 근무 형태와 개인의 생체리듬 적응 정도에 따라 그 영향이 달라질 수 있는 것으로 보고되고 있다[12].

한편, 야간전담제도가 도입·확대되기 이전의 연구들은 주로 교대근무의 부정적 영향에 초점을 두어 왔으나[13-15], 2022년 제도가 확대된 이후 야간전담근무와 3교대근무 간 생리적 적응 양상과 수면, 피로 및 업무성과의 차이를 비교한 연구는 제한적이다. 이에 본 연구는 동일 기관 간호사를 대상으로 야간근무형태(3교대근무군과 야간전담근무군)에 따른 수면의 질, 불면증, 만성피로 및 업무성과의 차이와 상관관계를 규명하고자 한다. 이를 통해 교대근무가 간호사의 생리적 적응과 직무 수행에 미치는 영향을 간호학적 근거로 제시하고, 향후 교대근무 환경에서 간호사의 생리적 건강을 증진시키기 위한 중재 전략 개발의 기초자료를 제공하고자 한다.

### 2. 연구의 목적

본 연구는 동일 기관에서 근무하는 간호사를 대상으로 설문 시점의 야간근무형태(3교대 근무군과 야간전담근무군)에 따른 수면의 질, 불면증, 만성피로 및 업무성과 간의 상관관계를 확인한 후, 업무성과에 대한 독립적인 영향요인을 규명하는 횡단적 서술적 조사연구이다. 구체적인 목적은 다음과 같다.

1) 3교대근무군과 야간전담근무군 간호사의 일반적 특성을 비교한다.

2) 3교대근무군과 야간전담근무군 간호사의 야간근무 관련 특성을 비교한다.

3) 야간근무형태(3교대근무군과 야간전담근무군)에 따른 수면의 질, 불면증, 만성피로 및 업무성과의 차이를 분석한다.

4) 야간근무형태(3교대근무군과 야간전담근무군)에 따른 수면의 질, 불면증, 만성피로 및 업무성과 간의 상관관계를 규명한다.

5) 업무성과에 영향을 미치는 요인을 규명한다.

## 연구 방법

### 1. 연구 설계

본 연구는 동일 기관 간호사를 대상으로 야간근무형태(야간전담근무군과 3교대근무군)에 따른 수면의 질, 불면증, 만성피로 및 업무성과의 차이를 비교하고, 변수 간 상관관계를 분석하며, 야간근무형태가 업무성과에 미치는 영향을 규명하기 위한 횡단적 서술적 조사연구이다. 본 연구는 관찰연구 보고지침인 Strengthening the Reporting of Observational Studies in Epidemiology (STROBE) 지침에 따라 보고하였다.

### 2. 연구 대상

본 연구의 대상자는 가톨릭대학교 서울성모병원에서 야간전담제를 운영하고 있는 병동에 근무하는 간호사로, 연구의 목적을 이해하고 자발적으로 참여에 동의한 자이다.

야간전담근무는 오후 10시에 시작하여 익일 오전 7시 30분에 종료되며, 기관 운영 기준에 따라 월 14일의 야간근무를 수행하였다. 이에 대한 보상으로 월 2일의 추가 야간근무 휴가가 부여되었다. 또한 야간전담 간호사는 2~3개월 이내의 연속 야간근무 후 3교대 근무로 복귀하는 조건에 동의하고 이를 희망하여 해당 야간근무형태로 배치된 간호사이다. 본 연구에서는 설문 시점의 야간근무형태를 기준으로 3교대근무군과 야간전담근무군으로 구분하였다.

대상자에 포함된 간호사는 야간근무를 시작한 지 6개월 이상 경과한 간호사로 하였으며, 입사 후 6개월이 경과하지 않은 신입간호사는 야간근무 경험이 충분하지 않다고 판단되어 제외하였다.

자료수집을 위해 총 270부의 설문지를 배부하였으며, 268부가 회수되었다(회수율 99.3%). 이 중 응답이 불완전하거나 중복 응답이 확인된 4부를 제외하고 최종 264부를 분석에 사용하였다. 설문 시점 기준으로 3교대 근무군은 161명, 야간전담 근무군은 103명이었다.

### 3. 표본의 크기

본 연구의 표본 수는 G*Power 3.1.9.4 프로그램을 이용하여 산출하였다[16]. 집단 간 평균 비교를 위한 independent t-test를 기준으로 효과크기(effect size) .50, 유의수준(α) .05, 검정력(1-β) .95를 적용하였다. 그 결과 최소 필요 표본 수는 총 210명으로 산출되었으며, 탈락률 20%를 고려하여 최종 목표 표본 수를 270명으로 설정하였다.

### 4. 연구 도구

#### 1) 3교대 간호사와 야간전담 간호사의 일반적 특성 및 야간 근무 특성

일반적 특성으로는 연령, 성별, 결혼 상태, 자녀 유무, 최종 학력, 근무 부서 및 임상경력을 조사하였다. 야간근무 관련 특성으로는 야간근무 시 평균 담당 환자 수, 병동 내 야간전담 간호사 비율, 야간근무 시 다른 근무조와 동수 근무 여부를 조사하였다. 직무만족도와 야간전담제 만족도는 전반적인 만족 수준을 단일 문항으로 측정하였다. 각 문항은 ‘전혀 만족하지 않는다(0점)’에서 ‘매우 만족한다(10점)’까지의 11점 Likert 척도로 구성되었으며, 점수가 높을수록 만족도가 높은 것을 의미한다.

#### 2) 수면의 질 지수(Korean version of the Pittsburgh Sleep Quality Index, PSQI-K)

수면의 질은 Buysse 등[17]이 개발한 피츠버그 수면의 질 지수(Pittsburgh Sleep Quality Index)를 Cho [18]가 번역하여 타인에 의해 영향을 받는 1문항을 제외하고 수정, 구성한 한국어판 피츠버그 수면의 질 지수(PSQI-K) 도구를 저자의 허락을 얻어 사용하였다. PSQI-K는 주관적 수면의 질, 수면 잠복기, 수면 기간, 습관적 수면 효율성, 수면 방해, 수면제 사용 및 주간 기능 장애의 7개 영역, 총 18문항으로 구성되어 있다. 각 영역은 PSQI-K 산출 지침에 따라 0점에서 3점까지 점수화되며, 7개 영역 점수를 합산하여 총점을 산출한다. 총점 범위는 0점에서 21점이며, Buysse 등[17]이 제시한 기준에 따라 5점을 초과할 경우 수면의 질이 저하된 것으로 해석한다. 점수가 높을수록 수면의 질이 낮음을 의미한다. 도구 개발 당시 신뢰도는 Cronbach's α .83이었고 선행연구의 신뢰도는 Cronbach’s α .74였으며 본 연구에서 측정한 신뢰도는 Cronbach’s α .79였다.

#### 3) 불면증 척도(Insomnia Severity Index)

불면증은 Bastien 등[19]이 개발한 Insomnia Severity Index를 Cho 등[20]이 번역·타당화한 한국어판 도구를 사용하여 측정하였다. 도구 사용에 대한 승인을 받은 후 적용하였다. 본 도구는 총 7문항으로 구성되어 있으며, 각 문항은 ‘전혀 없다(0점)’에서 ‘매우 심하다(4점)’까지의 5점 Likert 척도로 측정된다. 총점 범위는 0점에서 28점이며, 점수가 높을수록 불면증의 중증도가 높은 것을 의미한다. 점수 해석 기준은 0~7점은 정상, 8~14점은 경도 불면증, 15~21점은 중등도 불면증, 22~28점은 중증 불면증으로 구분한다. Cho 등[20]의 연구에서 보고된 신뢰도(Cronbach’s α)는 .92였으며, 본 연구에서의 Cronbach’s α는 .85였다.

#### 4) 만성피로도

만성피로도는 Kim 등[14]의 연구에서 사용된 도구를 도구 사용 승인을 받은 후 적용하였다. 만성피로는 충분한 수면이나 휴식을 취하였음에도 불구하고 에너지가 고갈된 상태와 지속적인 피곤함을 느끼는 상태를 의미한다. 본 도구는 총 10문항으로 구성되어 있으며, 각 문항은 ‘전혀 아니다(1점)’에서 ‘항상 그렇다(5점)’까지의 5점 Likert 척도로 측정된다. 총점 범위는 10점에서 50점이며, 점수가 높을수록 만성피로 수준이 높음을 의미한다. Kim 등[14]의 연구에서 보고된 신뢰도(Cronbach’s α)는 .89였으며, 본 연구에서의 Cronbach’s α는 .91이었다.

#### 5) 간호업무성과

간호업무성과는 Ko 등[21]이 개발한 간호업무성과 측정도구를 사용하였다. 연구 수행 전 원저자로부터 도구 사용 승인을 받았다. 본 도구는 총 17문항으로 구성되어 있으며, 업무성과, 업무수행태도, 업무수준 향상, 간호과정 적용의 4개 하위요인을 포함한다. 각 문항은 ‘전혀 그렇지 않다(1점)’에서 ‘매우 그렇다(5점)’까지의 5점 Likert 척도로 측정되며, 문항 평균 점수를 산출하여 사용하였다. 평균 점수가 높을수록 간호업무성과 수준이 높음을 의미한다. 도구 개발 당시 신뢰도(Cronbach’s α)는 .92였으며[21], 본 연구에서의 Cronbach’s α는 .91이었다.

### 5. 자료 수집

자료수집은 2023년 11월 4일부터 11월 19일까지 자가보고식 설문조사를 통해 이루어졌다. 연구 수행에 앞서 간호부의 승인을 받았으며, 부서 내 게시판에 연구 참여 모집 공고를 게시하였다. 이후 연구자가 각 병동을 방문하여 연구 목적과 절차를 설명하였다. 설문지는 익명성을 보장하기 위하여 간호 스테이션에 비치하였으며, 참여를 희망하는 간호사가 연구 내용을 충분히 이해한 후 자발적으로 설문에 응답하도록 하였다. 응답이 완료된 설문지는 부서와 개인 식별이 불가능하도록 간호사가 직접 밀봉하여 지정된 장소에 제출하도록 하여 익명성과 비밀보장을 유지하였다. 소정의 사례품은 자율적으로 수령하도록 하였고 회수된 설문지는 책임연구자가 수거하였다.

### 6. 자료 분석

수집된 자료는 IBM SPSS 프로그램 version 30.0 (IBM Corp., Armonk, NY, USA)을 이용하여 분석하였다.

간호사의 일반적 특성과 근무 특성은 빈도, 백분율, 평균 및 표준편차로 제시하였다. 교대근무 형태에 따른 일반적 특성 및 근무 특성의 차이는 independent t-test와 chi-square test로 분석하였으며, 본 연구에서 주요 변수의 정규성을 검정한 결과, Kolmogorov-Smirnov 검정과 Shapiro-Wilk 검정에서 모두 유의한 결과(*p* < .001)가 나타나 정규분포를 따르지 않는 것으로 확인되었다. 이에 따라 집단 간 차이 분석에는 비모수 검정인 Mann–Whitney U test를 사용하였으며, Mann–Whitney U test에서 유의한 차이를 보인 변수에 대해 효과크기 r을 |Z|/√N으로 산출하였다. 불면증 중증도와 같은 범주형 변수는 chi-square test를 이용하였다.

업무성과와 관련된 요인을 확인하기 위해 전체 대상자를 대상으로 일반적 특성, 야간근무 관련 특성 및 주요 연구변수에 따른 업무성과의 차이를 Mann–Whitney U test와 Kruskal–Wallis test로 분석하고 유의한 변수는 Bonferroni correction로 사후분석 하였다. 연속형 변수 간 상관관계는 주요 변수의 비정규성을 고려하여 Spearman의 순위상관계수(Spearman’s rank correlation coefficient)를 이용하여 분석하였다. 이후 야간근무형태와 업무성과 간의 독립적 관련성을 확인하기 위해 위계적 다중회귀분석(hierarchical multiple regression analysis)을 실시하였다. 위계적 다중회귀분석 시행 전 회귀모형의 가정을 검토하였다. 잔차의 정규성은 회귀 표준화 잔차의 histogram과 normal P–P plot을 통해 확인하였으며, 선형성과 등분산성은 표준화 예측값과 표준화 잔차의 산점도를 이용하여 확인하였다. 이상값 및 영향점은 표준화 잔차, studentized deleted residual, leverage 및 Cook's distance를 이용하여 검토하였다. 변수 투입 순서는 선행연구와 본 연구의 목적을 근거로 설정하였으며, 1단계에서는 단변량분석 및 상관분석에서 업무성과와 관련성이 확인된 변수들을 투입하였다. 2단계에서는 주요 독립변수인 야간근무형태를 추가 투입하여 야간근무형태가 업무성과의 설명력 증가에 추가적으로 기여하는지를 확인하였다.

### 7. 윤리적 고려

본 연구는 가톨릭대학교 서울성모병원 기관생명윤리위원회의 승인(IRB No. KC23QISI0561)을 받은 후 시행되었다. 간호사에게 연구 목적과 절차, 익명성 보장, 자료의 비밀 유지 및 연구 목적 외 사용 금지에 대해 설명하였으며, 설문지 배부 이후에도 참여를 원하지 않을 경우 언제든지 철회할 수 있음을 안내하였다. 설문지는 무기명으로 수집되었으며, 개인을 식별할 수 있는 정보는 일체 수집하지 않았다. 수집된 자료는 연구 목적에 한해 통계적으로 분석되었고, 보안이 유지된 파일로 저장하여 연구자만 접근할 수 있도록 관리하였다. 자료는 연구 종료 후 3년간 보관한 뒤 폐기할 예정이다. 연구 참여자에게는 소정의 답례품을 제공하였다.

## 연구 결과

### 1. 간호사의 근무 형태에 따른 일반적 특성의 차이

야간근무형태에 따른 간호사의 일반적 특성 차이를 분석한 결과, 3교대근무군과 야간전담근무군 간 연령, 성별, 결혼 상태, 최종 학력, 근무 부서 및 임상경력에 따라 유의한 차이가 없어 일반적 특성의 동질성이 확보되었다(Table 1). 총 264명 간호사 중 설문 시점 기준으로 3교대근무군은 161명(61.0%), 야간전담근무군은 103명(39.0%)이었다. 간호사의 평균 연령은 27.9 ± 3.1세였으며, 26~30세가 149명(56.4%)으로 가장 높은 비율을 차지하였다. 성별은 여성이 253명(95.8%)으로 대부분을 차지하였다. 결혼 상태는 미혼이 235명(89.0%)으로 가장 많았으며, 교육 수준은 학사학위가 243명(92.0%)으로 가장 높은 비율을 보였다. 근무 부서는 내과계 병동이 126명(47.7%)으로 가장 많았고, 외과계 병동이 99명(37.5%), 중환자실이 39명(14.8%)이었다. 임상경력은 평균 57.27 ± 36.52개월이었으며, 25~60개월이 123명(46.6%)으로 가장 높은 분포를 나타냈다.

### 2. 야간전담 간호사와 3교대 간호사의 근무 형태 특성

야간전담 간호사와 3교대 간호사의 근무 형태 특성을 비교한 결과 근무조 당 담당 환자 수는 3교대 간호사 9.85 ± 4.17명, 야간전담 간호사 10.73 ± 3.95명으로 나타났으나 통계적으로 유의한 차이는 없었다. 병동 내 야간전담 간호사 비율은 3교대 간호사 12.3 ± 5.9%, 야간전담 간호사 14.9 ± 5.5%로, 야간전담 간호사가 유의하게 높았다(t = -3.45, *p* = .001). 월 평균 야간근무 일수는 3교대 간호사 4.21 ± 1.48일, 야간전담 간호사 14.00 ± 0.86일로, 야간전담 간호사가 유의하게 많았다(t = -67.9, *p* < .001). 야간근무 시 다른 근무조와 동수 근무 여부에 대해서는 전체 간호사 중 74.2%가 야간근무 시 인력을 축소하여 운영한다고 응답하였고 두 집단 간 유의한 차이를 보이지 않았다. 직무만족도는 10점 만점에 3교대근무군 5.57 ± 1.90점, 야간전담근무군 5.47 ± 1.94점으로 차이가 없었고, 야간전담제 만족도 역시 3교대근무군 6.53 ± 2.11점, 야간전담근무군 6.74 ± 2.05점으로 유의한 차이가 없었으며, 직무만족도 수준의 분포나 야간전담제 만족도 수준의 분포도 집단 간 차이가 없었다(Table 2).

### 3. 3교대 간호사와 야간전담 간호사의 수면의 질, 불면증, 만성피로 및 업무성과 비교

교대근무 형태에 따른 차이를 분석한 결과, 총 수면의 질(PSQI-K) 점수는 3교대 간호사 8.72 ± 3.01점, 야간전담 간호사 8.94 ± 2.97점으로 나타났으며, PSQI-K의 하위 영역(주관적 수면의 질, 수면 잠복기, 수면 지속시간, 수면 효율성, 수면 방해, 수면제 사용, 주간 기능장애)을 포함하여 집단 간 유의한 차이는 나타나지 않았다. 불면증 총점은 3교대 간호사 9.69 ± 5.14점, 야간전담근무 간호사 11.12 ± 5.40점으로 나타났으며, 야간전담 간호사에서 유의하게 높았다(Z = -2.16, *p* = .031, r = .13). 그러나 불면증 중증도 범주에 따른 분포에서는 두 집단 간 유의한 차이가 나타나지 않았다.

만성피로는 3교대 간호사 30.92 ± 6.66점, 야간전담 간호사 31.80 ± 6.83점으로 나타났으며, 집단 간 유의한 차이는 없었다. 업무 성과 또한 3교대 간호사 3.92 ± 0.39점, 야간전담 간호사 4.02 ± 0.44점으로 나타났으나, 유의한 차이는 없었다(Table 3).

### 4. 일반적 특성에 따른 업무성과와 만성피로의 차이

업무성과는 교육수준, 임상경력, 직무만족도와 유의한 관련성을 보였다. 교육수준에 따라 업무성과에 유의한 차이가 있었으며, 석사 학위 이상 집단의 업무성과가 학사 학위 집단보다 높았다(Z = 3.04, *p* = .002). 또한 임상경력(rho = .15, *p* = .017)과 직무만족도(rho = .32, *p* < .001)는 업무성과와 유의한 양의 상관관계를 보였다. 직무만족도를 구간화한 분석에서도 세 집단 간 업무성과에 유의한 차이가 있었고, Bonferroni 보정 사후분석 결과 7~10점군이 0~3점군 및 4~6점군보다 업무성과가 유의하게 높았다(H = 20.26, *p* < .001, a,b < c) (Appendix 1).

만성피로는 나이, 임상경력, 직무만족도, 야간전담제 만족도와 유의한 관련성을 보였다. 나이에 따라 만성피로에 유의한 차이가 있었으며, 25세 이하 집단이 26~30세 및 31세 이상 집단보다 만성피로가 유의하게 낮았다(H = 7.50, *p* = .024, a < b,c). 임상경력은 만성피로와 유의한 양의 상관관계를 보였으며(rho = .13, *p* = .040), 구간별 유의한 차이가 없었다. 직무만족도(rho = -.35, *p* < .001)와 야간전담제 만족도(rho = -.24, *p* < .001)는 만성피로와 유의한 음의 상관관계를 보였다. Bonferroni 보정 사후분석 결과, 직무만족도는 7~10점군이 다른 군보다 만성피로가 유의하게 낮았고(H = 35.50, *p* < .001, a,b > c), 야간전담제 만족도는 0~3점군이 다른 군보다 만성피로가 유의하게 높았다(H = 18.33, *p* < .001, a > b,c)(Appendix 2).

### 5. 간호사의 수면의 질, 불면증, 만성피로, 업무성과, 직무만족도, 야간전담제 만족도 및 임상경력 간의 상관관계

3교대근무 간호사에서는 수면의 질, 불면증, 만성피로 및 만족도 변수가 업무성과와 유의하게 관련되었으나, 야간전담 간호사에서는 직무만족도만이 업무성과와 유의한 관련성을 보였다. Spearman 상관관계 분석 결과, 3교대 간호사에서 업무성과는 PSQI-K와 유의한 음의 상관관계를 보였으며(rho = -.20, *p* = .011), 불면증(rho = -.24, *p* = .002) 및 만성피로(rho = -.26, *p* < .001)와도 유의한 음의 상관관계를 보였다. 즉, 수면의 질이 낮고, 불면증과 만성피로가 높을수록 업무성과가 낮은 경향을 보였다. 반면 3교대 간호사 업무성과는 야간전담제 만족도(rho = .20, *p* = .013), 직무만족도(rho = .35, *p* < .001) 및 임상경력 (rho = .35, *p* < .001)과 유의한 양의 상관관계를 보였다. 또한 업무성과와 유의한 양의 상관관계를 보였다. 야간전담 간호사에서는 업무성과가 직무만족도와는 유의한 양의 상관관계를 보였으나(rho = .27, *p* = .006), PSQI-K, 불면증, 만성피로, 야간전담제 만족도, 임상경력과 상관관계를 보이지 않았다.

수면 관련 변수 간의 관계를 보면, 3교대 간호사에서 PSQI-K는 불면증(rho = .67, *p* < .001) 및 만성피로(rho = .37, *p* < .001)와 유의한 양의 상관관계를 보였고, 불면증 역시 만성피로와 유의한 양의 상관관계를 보였다(rho = .43, *p* < .001). 야간전담 간호사에서도 PSQI-K는 불면증(rho = .67, *p* < .001) 및 만성피로(rho = .26, *p* = .008)와 유의한 양의 상관관계를 보였으며, 불면증은 만성피로와 유의한 양의 상관관계를 보였다(rho = .43, *p* < .001). 직무만족도와 야간전담제 만족도는 3교대군 (rho = .48, *p* < .001)과 야간전담군(rho = .48, *p* < .001) 모두에서 유의한 양의 상관관계를 보였다(Table 4).

### 6. 업무성과에 영향을 미치는 요인에 대한 위계적 다중회귀분석

업무성과에 영향을 미치는 요인을 분석하기 위하여 위계적 회귀분석을 시행하였다. 위계적 다중회귀분석을 실시하기에 앞서 회귀모형의 기본 가정을 검토하였다. 다중공선성을 확인한 결과, 공차한계(tolerance)는 .72~.96으로 .10이상이었으며, 분산팽창지수(variance inflation factor [VIF])는 1.04~1.38로 10 미만으로 나타나 다중공선성의 문제는 없었다. 최종 회귀모형의 Durbin–Watson 통계량은 2.02-2.04로 나타나 잔차의 독립성 가정이 충족되었다. 또한 표준화 잔차의 P-P plot을 통해 잔차의 정규성을 확인하였고, 표준화 예측값과 표준화 잔차의 산점도를 통해 선형성과 등분산성 가정을 확인하였다. Cook’s distance로 이상치 및 영향력이 큰 관측치를 확인한 결과 회귀모형에 영향을 줄 만한 사례는 없었다.

위계적 다중회귀분석을 위해 Model I에서는 교육수준, 직무만족도, 불면증, 임상경력, 만성피로를 투입하였고, Model II에서는 야간근무형태를 추가로 투입하였다. Model I의 투입 변수 중 PSQI-K와 불면증은 높은 상관관계로 인한 다중공선성 가능성을 고려하여 군간 유의한 차이를 보인 불면증을 수면 관련 변수로 투입하고 PSQI-K는 제외하였다.

Model I은 통계적으로 유의하였으며(F = 8.88, *p* < .001), 업무성과에 대한 설명력은 15.0% (R² = .15, adjusted R² = .13)였다. Model I에서 업무성과에 유의한 영향을 미치는 요인은 교육수준(β = .14, *p* = .028)과 직무만족도(β = .27, *p* < .001)이었다.

야간근무형태를 추가로 투입한 Model II 역시 통계적으로 유의하였다(F = 8.65, *p* < .001). 최종모형에서 업무성과에 유의한 영향을 미치는 요인은 석사학위이상의 교육수준(β = .13, *p* = .042), 직무만족(β = .27, *p* < .001)과 야간근무형태(β = .15, *p* = .011)였고 설명력은 17.0% (R² = .17, adjusted R² = .15) 였으며, 야간근무형태 추가에 따른 설명력 증가분은 2%p였다(ΔR² = .02). 즉, 직무만족이 높을수록 업무성과가 높았으며, 야간전담 근무자의 업무성과가 높았다. 반면 임상경력, 불면증 및 만성피로는 최종모형에서 업무성과에 유의한 영향을 미치지 않았다(Table 5).

## 논의

본 연구는 동일 기관 간호사를 대상으로 야간근무형태(3교대근무군과 야간전담근무군)에 따른 수면의 질, 불면증, 만성피로 및 업무성과의 차이와 상관관계를 확인하고, 업무성과에 영향을 미치는 요인을 규명하고자 수행되었다. 「간호인력 야간근무 가이드라인」에서는 야간전담 간호사의 월 야간근무를 15일 이내, 연속 야간근무를 3일 이하로 제한하고, 건강권 보호를 위해 야간전담근무의 연속기간을 원칙적으로 3개월 이하로 제시하고 있어[22], 야간전담근무와 3교대근무는 혼용되어 운영되고 있는 실정이다. 본 연구에서 전체 간호사의 PSQI-K 평균 점수는 3교대와 야간전담근무 모두 절단점인 5점보다 높아 수면의 질이 낮은 수준으로 나타났다. 이는 선행연구[23,24]에서 보고된 PSQI-K점수보다 높은 수준으로, 야간근무를 수행하는 간호사들의 수면 건강이 취약한 상태임을 시사한다. 그러나 야간근무형태에 따른 총 PSQI-K 점수와 하위영역에서는 두 군이 유의한 차이가 나타나지 않아 야간전담근무가 야간노출 빈도는 높지만 주관적 수면의 질 자체에서는 3교대근무와 큰 차이를 보이지 않을 가능성을 시사한다. 야간전담근무는 일정 기간 동안 동일한 야간근무형태가 유지되기 때문에 교대주기가 반복적으로 변화하는 3교대근무에 비해 생리적 적응이 상대적으로 용이할 수 있다[25]. 다만 본 연구 결과만으로 야간전담근무가 수면의 질에 보호적 효과를 가진다고 해석하기는 어려우며, 근무 지속 기간, 연속 야간근무 일수, 회복시간 등을 함께 고려한 추가 연구가 필요하다.

불면증 총점은 야간전담 간호사가 유의하게 높았는데 야간전담 간호사의 월 평균 야간근무 일수가 3교대 간호사보다 유의하게 많은 것과 관련되어 보이며 반복적이고 집중적인 야간노출이 불면 증상 증가와 연관될 수 있다는 선행연구[3]의 결과와도 맥락을 같이한다. 즉, 고정된 야간근무가 일정한 근무패턴을 제공하더라도 생체리듬의 완전한 적응을 보장하지는 않으며, 오히려 불면 증상 증가와 관련될 수 있음을 보여준다. 선행연구에서도 야간근무는 멜라토닌 분비 억제 및 수면-각성 리듬의 변화를 유발하여 수면장애를 증가시키는 것으로 보고된 바 있다[1,26]. 간호사의 수면의 질 향상과 환자 안전을 위해 야간전담근무자의 월 야간근무 일수와 연속 야간근무 일수 또한 적정성 검토가 필요할 것으로 생각된다.

상관관계 분석에서는 3교대 간호사에서 수면의 질, 불면증 및 만성피로가 업무성과와 유의한 음의 상관관계를 나타낸 반면, 야간전담 간호사에서는 이러한 관련성이 나타나지 않았다. 이는 교대근무의 불규칙성이 수면 문제를 통해 업무 수행 능력과 관련될 수 있음을 의미하며, 교대근무 간호사에서 수면장애와 직무 수행 간의 밀접한 관련성을 보고한 선행연구와 일치한다[27]. 반면, 야간전담근무는 일정한 근무패턴에 반복적으로 노출되므로 3교대근무에 비해 생리적 적응이 상대적으로 용이할 수 있으며[28], 이러한 특성이 수면 관련 요인과 업무성과 간 관련성을 약화시켰을 가능성이 있다. 그러나 야간전담 간호사에서 수면 관련 요인과 업무성과 간의 유의한 상관관계가 나타나지 않았다는 결과는 야간전담근무가 업무성과에 긍정적 영향을 미친다는 의미보다, 업무성과가 수면 요인 외의 직무·조직적 요인에 의해 더 크게 설명될 가능성을 보여주는 것으로 해석할 필요가 있다.

위계적 다중회귀분석 최종 모형에서는 교육수준, 직무만족도, 야간근무형태가 업무성과에 유의한 영향을 미치는 요인으로 확인되었으며, 특히 직무만족도가 가장 큰 영향력을 보였다. 교육수준은 Model I과 야간근무형태를 추가한 최종모형 모두에서 업무성과의 유의한 영향요인으로 확인되었다. 이는 교육수준이 높을수록 간호업무 수행에 필요한 지식과 전문적 역량이 향상되어 업무성과와 긍정적으로 관련될 가능성을 시사한다. 또한 직무만족도와 야간근무형태 역시 최종모형에서 유의한 영향요인으로 나타났다. 이러한 결과는 업무성과가 단순한 생리적 요인보다는 직무만족도와 같은 조직적·심리적 요인의 영향을 더 크게 받을 수 있음[29]을 보여준다.

또한 위계적 회귀 최종 모형에서 불면증이 영향 요인으로 확인되지 않았는데, 불면증은 업무성과와 단순 상관관계 수준에서는 관련될 수 있으나, 직무만족도, 임상경력 및 야간근무형태를 함께 고려하여 통제했을 때 업무성과에 대한 독립적인 설명력은 제한적임을 시사한다. 따라서 불면증이 업무성과에 미치는 영향은 직접적이기보다 피로, 직무만족도 또는 근무환경 요인을 통해 간접적으로 나타날 가능성이 있으며[10,12], 추후 연구에서는 이들 변수 간의 매개 또는 조절 관계를 확인할 필요가 있다.

특히 직무만족도는 모든 단계에서 일관되게 유의한 영향요인으로 나타났는데, 이는 직무만족이 높을수록 업무몰입과 직무 수행 능력이 향상된다는 선행연구와 일치하는 결과이다[30]. 교육수준은 Model I과 Model II 에서 업무성과에 영향을 미치는 유의한 영향 요인이었지만 본 연구에서 석사 이상 대상자의 비율이 상대적으로 낮았기 때문에, 교육수준의 영향을 일반화할 때에는 신중한 해석이 필요하다. 본 연구 결과는 야간전담근무가 반드시 수면 건강에 유리한 야간근무형태임을 의미하지는 않는다. 실제로 본 연구에서 야간전담근무군은 3교대근무군보다 불면증 수준이 유의하게 높았다. 그럼에도 불구하고 최종 회귀모형에서 야간근무형태가 업무성과와 유의한 양의 관련성을 보인 것은, 업무성과가 수면 관련 요인만으로 설명되지 않으며 야간근무형태의 안정성, 업무 예측 가능성, 직무만족도 및 근무 적응과 같은 직무·조직적 요인의 영향을 함께 받을 수 있음을 시사한다[10,11].

또한 야간전담근무는 자발적 선택에 의해 이루어지는 경우가 많아 직무 적합성과 직무 몰입 수준이 상대적으로 높을 수 있으며, 이러한 요인은 수면 문제와 업무성과 간의 직접적인 연관성을 약화시켰을 가능성이 있다[25]. 특히 야간전담 간호사를 대상으로 한 포커스 그룹 연구에서는, 고정된 근무 형태가 업무의 연속성과 일관성을 유지시키고 반복 수행을 통한 업무 숙련과 역할 적응을 촉진하여 예측 가능한 생활 리듬 형성에 기여하는 것으로 보고되었다[25].

야간전담근무 제도는 다른 간호사의 야간근무 부담을 감소시키고 개인의 생활 패턴을 보다 안정적으로 유지할 수 있게 하는 긍정적인 측면이 있는 것으로 보고되고 있다[10]. 선행연구에서도 야간전담근무의 장점으로 규칙적인 생활 패턴, 휴일 증가, 야간수당, 비교적 조용한 근무환경 등이 제시되었으며[14], 제도 인식 조사에서도 높은 만족도와 근무 지속 의사가 보고된 바 있다[13]. 다만 본 연구 결과는 야간전담근무 제도를 업무성과 향상을 위한 직접적인 대안으로 제시하기보다는, 야간전담근무의 잠재적 역할을 직무만족도, 근무환경, 수면 건강 지원과 함께 종합적으로 검토할 필요가 있음을 시사한다.

야간전담근무는 일정한 근무패턴에 반복적으로 노출되므로 3교대근무에 비해 근무시간과 업무환경의 예측 가능성이 높을 수 있으며, 이러한 특성이 수면 관련 요인과 업무성과 간 관련성을 약화시켰을 가능성이 있다[11]. 다만 야간전담근무가 불면증 부담을 동반할 수 있으므로, 제도적 효과를 높이기 위해서는 수면위생 교육, 근무표 설계, 충분한 휴식 보장 등 수면 건강 지원이 함께 이루어져야 한다.

그러나 본 연구의 위계적 회귀분석에서는 최종모형의 설명력이 17.0% (R² = .17, adjusted R² = .15)로서, 확인된 예측요인들의 영향력은 제한적인 수준으로 해석할 필요가 있다. 특히 야간근무형태는 다른 변수들을 통제한 후 유의한 관련성을 보였으나, 단변량 분석에서는 업무성과의 유의한 차이가 확인되지 않았고 설명력 증가분도 작았으므로, 야간전담근무가 업무성과에 미치는 영향을 단정적으로 해석하는 데에는 세심한 주의가 요구된다.

본 연구는 단일 기관 간호사를 대상으로 수행된 횡단적 연구로, 연구 결과를 일반화하는 데 제한이 있다. 표본 수 산정 시 집단 간 평균 비교를 위한 independent t-test를 기준으로 하였으나, 실제 분석에서는 주요 변수의 정규성 가정이 충족되지 않아 비모수 검정을 적용하였다. 이는 표본수 산정 기준과 실제 분석 방법 간 차이로 볼 수 있으므로, 결과 해석 시 고려할 필요가 있다. 또한 본 연구는 자기보고식 설문을 사용하였으며, 멜라토닌, 코르티솔, 활동량, 수면다원검사 등 생리적 지표를 직접 측정하지 않았다. 따라서 근무형태에 따른 생체리듬 변화, 생리적 적응 또는 재동조 여부를 직접적으로 해석하는 데에는 제한이 있다. 또한, 직무만족도를 단일 문항으로 측정하여 다차원적인 직무만족 개념을 충분히 반영하지 못했을 가능성과 더불어 업무성과에 영향을 미치는 다양한 조직 및 환경 요인을 충분히 반영하지 못하여 설명력이 제한적일 수 있다. 그럼에도 불구하고 본 연구는 야간전담근무 제도가 확대 적용되고 있는 시점에서 야간전담 간호사와 3교대 간호사의 수면 건강과 업무성과를 비교하고 관련 요인을 탐색함으로써 향후 제도 운영과 간호사의 건강 보호를 위한 기초자료를 제공했다는 점에서 의의가 있다.

## 결론 및 제언

본 연구는 3교대 간호사와 야간전담 간호사의 수면의 질, 불면증, 만성피로, 업무성과 및 직무만족도 간의 관계를 확인하였다. 연구 결과, 3교대 간호사에서는 수면의 질이 낮고 불면증과 만성피로가 높을수록 업무성과가 낮았으며, 야간전담제 만족도와 직무만족도가 높을수록 업무성과가 높았다. 반면 야간전담 간호사에서는 수면 관련 요인과 업무성과 간의 유의한 관련성이 나타나지 않았다. 전체 대상자를 대상으로 한 위계적 회귀분석에서는 교육수준, 직무만족도와 야간근무형태가 업무성과의 유의한 영향요인으로 확인되었다. 그러나 야간근무형태는 다른 변수들을 통제한 후에만 업무성과와 유의한 관련성을 보였고, 최종 회귀모형의 설명력과 근무형태 추가에 따른 설명력 증가분은 제한적이었으므로, 야간전담근무가 업무성과에 미치는 영향을 단정적으로 해석하는 데에는 주의가 필요하다. 따라서 간호사의 업무성과 향상을 위해서는 수면 건강 관리와 함께 직무만족도 향상을 위한 조직적 전략이 병행되어야 한다. 추후 연구에서는 종단연구를 통해 수면문제, 만성피로, 직무만족도, 야간근무형태 및 업무성과 간의 인과관계를 확인하고, 야간근무형태별 업무성과 영향요인을 보다 구체적으로 규명할 필요가 있다.

## Figures and Tables

**Table 1.** General Characteristics of Nurses According to Work Type (N = 264)

Characteristics	Categories	Total (n = 264)	3-shift (n = 161)	Fixed-night-shift (n = 103)	χ^2^ or t	*p*
Age (years)		27.9 ± 3.1	28.1 ± 3.3	27.4 ± 2.8	1.94	.054
	≤ 25	61 (23.1)	37 (23.0)	24 (23.3)	2.19	.147
	26–30	149 (56.4)	85 (52.8)	64 (62.1)		
	≥ 31	54 (20.5)	39 (24.2)	15 (14.6)		
Sex	Women	253 (95.8)	156 (96.9)	97 (94.2)	1.16	.348
	Men	11 (4.2)	5 (3.1)	6 (5.8)		
Marital status	Unmarried	235 (89.0)	139 (86.3)	96 (93.2)	3.05	.218
	Married (without children)	20 (7.6)	15 (9.3)	5 (4.9)		
	Married (with children)	9 (3.4)	7 (4.4)	2 (1.9)		
Education level	Bachelor's degree	243 (92.0)	149 (92.5)	94 (91.3)	0.14	.707
	≥ Master’s degree	21 (8.0)	12 (7.5)	9 (8.7)		
Department	Medical ward	126 (47.7)	75 (46.6)	51 (49.5)	0.65	.723
	Surgical ward	99 (37.5)	60 (37.3)	39 (37.9)		
	ICU	39 (14.8)	26 (16.1)	13 (12.6)		
Clinical career (months)		57.27 ± 36.52	60.53 ± 38.05	52.17 ± 33.54	1.82	.070
	＜25	44 (16.7)	27 (16.8)	17 (16.5)	2.72	.257
	25–60	123 (46.6)	69 (42.8)	54 (52.4)		
	> 60	97 (36.7)	65 (40.4)	32 (31.1)		

Values are presented as the mean ± standard deviation or n (%). ICU = Intensive care unit.

**Table 2.** Work Pattern Characteristics of Fixed-night-shift and 3-shift Nurses (N = 264)

Characteristics	Categories	Total (n = 264)	3-shift (n = 161)	Fixed-night-shift (n = 103)	χ^2^ or t	*p*
Number of patients in charge		10.20 ± 4.10	9.85 ± 4.17	10.73 ± 3.95	-1.71	.089
Percentage of fixed-night-shift nurses in the unit (%)		13.39 ± 5.89	12.31 ± 5.92	14.93 ± 5.52	-3.45	.001
Monthly night shifts		8.03 ± 4.95	4.21 ± 1.48	14.00 ± 0.86	-67.9	< .001
Night-shift staffing equal to other shifts	Same number	68 (25.8)	46 (28.6)	22 (21.4)	1.71	.191
	Decreased number	196 (74.2)	115 (71.4)	81 (78.6)		
Job satisfaction (0–10)		5.53 ± 1.91	5.57 ± 1.90	5.47 ± 1.94	0.41	.682
	Low (0–3)	46 (17.5)	25 (15.6)	21 (20.4)	1.06	.589
	Moderate (4–6)	126 (47.7)	78 (48.4)	48 (46.6)		
	High (7–10)	92 (34.8)	58 (36.0)	34 (33.0)		
Fixed night-shift system satisfaction (0–10)		6.61 ± 2.08	6.53 ± 2.11	6.74 ± 2.05	-0.80	.425
	Low (0–3)	23 (8.7)	17 (10.6)	6 (5.9)	2.09	.351
	Moderate (4–6)	77 (29.2)	44 (27.3)	33 (32.0)		
	High (7–10)	164 (62.1)	100 (62.1)	64 (62.1)		

Values are presented as the mean ± standard deviation or n (%).

**Table 3.** Differences in Sleep Quality, Insomnia, Chronic Fatigue, and Work Performance between 3-shift and Fixed-night-shift Nurses (N = 264)

Characteristics	Categories	Total (n = 264)	3-shift (n = 161)	Fixed-night-shift (n = 103)	χ^2^ or Z	*p*	*r*
PSQI-K	Total sleep quality	8.83 ± 2.98	8.72 ± 3.01	8.94 ± 2.97	-0.57	.566	.04
	Subjective quality	1.53 ± 0.59	1.50 ± 0.57	1.58 ± 0.62	-1.21	.228	.07
	Latency	1.77 ± 0.95	1.79 ± 0.92	1.73 ± 0.99	-0.46	.643	.03
	Duration	0.89 ± 1.01	0.89 ± 1.03	0.88 ± 0.99	-0.03	.976	.01
	Efficiency	0.68 ± 0.95	0.71 ± 0.98	0.62 ± 0.91	-0.62	.537	.04
	Disturbance	1.03 ± 0.46	1.01 ± 0.49	1.07 ± 0.43	-1.19	.235	.07
	Use of sleep medication	1.76 ± 0.60	1.80 ± 0.55	1.74 ± 0.64	-0.52	.607	.03
	Daytime dysfunction	1.15 ± 0.92	1.07 ± 0.90	1.26 ± 0.95	-1.46	.145	.09
Insomnia	Total insomnia	10.25 ± 5.28	9.69 ± 5.14	11.12 ± 5.40	-2.16	.031	.13
	0–7 (normal)	90 (34.1)	60 (37.3)	30 (29.1)	4.45	.217	
	8–14 (subthreshold)	116 (43.9)	72 (44.7)	44 (42.7)			
	15–21 (moderate severity)	53 (20.1)	26 (16.1)	27 (26.3)			
	22–28 (severe)	5 (1.9)	3 (1.9)	2 (1.9)			
Chronic fatigue		31.26 ± 6.73	30.92 ± 6.66	31.80 ± 6.83	-1.55	.121	.10
Work performance		3.96 ± 0.42	3.92 ± 0.39	4.02 ± 0.44	-1.48	.139	.09

Values are presented as the mean ± standard deviation or n (%). Z values were obtained from the Mann–Whitney U test; χ² values from the chi-square test. Effect size r was calculated for Mann–Whitney U test results using |Z|/√N. PSQI-K = Korean version of the Pittsburgh Sleep Quality Index.

**Table 4.** Correlations between Sleep Quality, Insomnia, Chronic Fatigue, Work Performance, Satisfaction, and Clinical Career among 3-shift and Fixed-night-shift Nurses (N = 264)

Variables	3-shift (n = 161)						Fixed-night-shift (n = 103)					
	PSQI-K	Insomnia	Chronic Fatigue	Clinical career	Job satisfaction	Fixed night-shift system satisfaction	PSQI-K	Insomnia	Chronic fatigue	Clinical career	Job satisfaction	Fixed night-shift system satisfaction
	rho (*ρ*)											
Insomnia	.67^**^ (< .001)	1					.67^**^ (< .001)	1				
Chronic Fatigue	.37^**^ (< .001)	.43^**^ (< .001)	1				.26^**^ (.008)	.43^**^ (< .001)	1			
Clinical career	.16^*^ (.046)	.15 (.055)	.16^*^ (.041)	1			.02 (.830)	.11 (.280)	.14 (.177)	1		
Job satisfaction	-.22^**^ (.004)	-.24^**^ (.002)	-.40^**^ (< .001)	-.15 (.055)	1		-.01 (.963)	-.09 (.358)	-.31^**^ (.001)	.07 (.466)	1	
Fixed night-shift system satisfaction	-.28^**^ (< .001)	-.25^**^ (.003)	-.27^**^ (< .001)	.20^*^ (.013)	.48^**^ (< .001)	1	.01 (.934)	-.16 (.107)	-.21^*^ (.037)	.14 (.161)	.48^**^ (< .001)	1
Work performance	-.20^*^ (.011)	-.24^**^ (.002)	-.26^**^ (< .001)	.35^**^ (< .001)	.35^**^ (< .001)	.20^*^ (.013)	.03 (.749)	.14 (.149)	-.08 (.397)	.18 (.064)	.27^**^ (.006)	-.05 (.636)

PSQI-K = Korean version of the Pittsburgh Sleep Quality Index; rho = Spearman’s rank correlation coefficients.

* *p* < .05;

** *p* < .01.

**Table 5.** Factors Influencing Work Performance (Hierarchical Multiple Regression) (N = 264)

Model	Variable	B	SE	β	t	*p*	Tolerance	VIF					
Ⅰ	(Constant)	3.77	0.16		23.05	<.001							
	Master’s degree education level (Bachelor's degree = 0)	0.22	0.10	.14	2.22	.028	.82	1.22					
	Job satisfaction	0.06	0.01	.27	4.43	<.001	.87	1.15					
	Insomnia	0.01	0.01	.01	0.14	.888	.81	1.23					
	Clinical career	0.01	0.01	.08	1.25	.211	.81	1.24					
	Chronic fatigue	−0.01	0.01	−.11	−1.64	.102	.72	1.38					
		F = 8.88, *p* < .001, R^2^ = .15, Adjusted R^2^ = .13, Durbin-Watson = 2.04											
Ⅱ	(Constant)	3.73	0.16		22.96	<.001							
	Master’s degree education level (Bachelor's degree = 0)	0.20	0.10	.13	2.04	.042	.81	1.24					
	Job satisfaction	0.06	0.01	.27	4.46	< .001	.87	1.15					
	Insomnia	−0.01	0.01	−.01	−0.16	.870	.80	1.25					
	Clinical career	0.01	0.01	.10	1.63	.105	.79	1.27					
	Chronic fatigue	−0.01	0.01	−.12	−1.72	.087	.72	1.38					
	Fixed night shift (3-shift = 0)	0.13	0.05	.15	2.56	.011	.96	1.04					
		F = 8.65, *p* < .001, R^2^ = .17, Adjusted R^2^ = .15											
		ΔR^2^ = .02, Durbin-Watson = 2.02											

SE = Standard error; VIF = Variance inflation factor. *p*-values were obtained using hierarchical multiple regression analysis. Dependent variable: Work performance. Model Ⅰ included education level, satisfaction with work, insomnia, clinical career, and chronic fatigue; Model Ⅱ included all variables in Model Ⅰ plus shift type. Reference groups were bachelor’s degree for education level and three-shift nurses for night shift type. Night shift type: 0 = 3-shift, 1 = fixed night shift; Education level: 0 = bachelor's degree, 1 = master’s degree.

## Appendix 1. Work Performance According to General Characteristics of Nurses (N =264)

**Table:**

Characteristics	Categories	Total	Work performance		
		n (%) or M ± SD	M ± SD	Z or H or Rho	*p* Bonferroni
Age (years)		27.9 ± 3.1	3.96 ± 0.42	.11^†^	.064
	≤ 25^a^	61 (23.1)	3.93 ± 0.38	3.03	.219
	26–30^b^	149 (56.4)	3.95 ± 0.43		
	≥ 31^c^	54 (20.5)	4.02 ± 0.42		
Sex	Women	253 (95.8)	3.96 ± 0.42	-0.85	.396
	Men	11 (4.2)	3.89 ± 0.33		
Marital status	Unmarried	235 (89.0)	3.95 ± 0.42	1.48	.477
	Married (no child)	20 (7.6)	4.00 ± 0.40		
	Married (with child)	9 (3.4)	4.13 ± 0.42		
Education level	University	243 (92.0)	3.93 ± 0.41	3.04	.002
	≥ Master's	21 (8.0)	4.22 ± 0.44		
Department	Medical Ward	126 (47.7)	3.97 ± 0.45	2.02	.364
	Surgical Ward	99 (37.5)	3.91 ± 0.37		
	ICU	39 (14.8)	4.05 ± 0.43		
Clinical career (months)		57.27 ± 36.52	3.96 ± 0.42	.15^†^	.017
	＜ 25^a^	44 (16.7)	3.87 ± 0.49	4.83	.089
	25–60^b^	123 (46.6)	3.95 ± 0.40		
	> 60^c^	97 (36.7)	4.01 ± 0.40		
All shift equal nurses	Same numbers	68(25.8)	4.00 ± 0.41	-0.64	.521
	Decrease numbers	196(74.2)	3.94 ± 0.42		
Job satisfaction		5.53 ± 1.92	3.96 ± 0.42	.32^†^	<.001
	0–3^a^	46(17.5)	3.79 ± 0.52	20.26	<.001 a,b < c^‡^
	4–6^b^	126(47.7)	3.90 ± 0.34		
	7–10^c^	92(34.8)	4.12 ± 0.41		
Fixed night-shift system satisfaction		6.61 ± 2.08	3.96 ± 0.42	.10^†^	.116
	0–3^a^	23(8.7)	3.86 ± 0.50	1.36	.507
	4–6^b^	77(29.2)	3.90 ± 0.37		
	7–10^c^	164(62.1)	4.00 ± 0.42		

Z = Mann-Whitney U test; H = Kruskal-Wallis test; ICU = Intensive care unit; M = Mean; SD = Standard deviation.

^†^Rho = Spearman’s rank correlation coefficients; ^‡^Post hoc pairwise comparisons were performed using Bonferroni correction.

## Appendix 2. Chronic Fatigue According to General Characteristics of Nurses (N = 264)

**Table:**

Characteristics	Categories	Total	Chronic fatigue		
		n (%) or M ± SD	M ± SD	Z or H or Rho	*p* Bonferroni
Age (years)		27.9 ± 3.1		.16^†^	.011
	≤ 25^a^	61 (23.1)	29.16 ± 6.90	7.50	.024 a < b,c^‡^
	26–30^b^	149 (56.4)	31.64 ± 6.66		
	≥ 31^c^	54 (20.5)	32.59 ± 6.27		
Sex	Women	253 (95.8)	31.27 ± 6.58	-0.09	.929
	Men	11 (4.2)	31.00 ± 10.02		
Marital status	Unmarried	235 (89.0)	31.31 ± 6.91	1.03	.599
	Married (no child)	20 (7.6)	31.55 ± 4.82		
	Married (with child)	9 (3.4)	29.44 ± 5.55		
Education level	University	243 (92.0)	31.25 ± 6.72	0.12	.905
	≥ Master's	21 (8.0)	31.38 ± 7.13		
Department	Medical Ward	126 (47.7)	30.60 ± 6.51	5.61	.061
	Surgical Ward	99 (37.5)	32.46 ± 6.07		
	ICU	39 (14.8)	30.33 ± 8.53		
Clinical career (months)		57.27 ± 36.52		.13^†^	.040
	＜ 25^a^	44 (16.7)	29.95 ± 6.54	2.75	.253
	25–60^b^	123 (46.6)	31.39 ± 6.67		
	> 60^c^	97 (36.7)	31.69 ± 6.87		
All shift equal nurses	Same numbers	68 (25.8)	30.37 ± 7.50	1.46	.227
	Decrease numbers	196 (74.2)	31.57 ± 6.43		
Job satisfaction		5.53 ± 1.92		-.35^†^	< .001
	0–3^a^	46 (17.5)	34.20 ± 7.55	35.50	< .001 a,b > c^‡^
	4–6^b^	126 (47.7)	32.63 ± 5.72		
	7–10^c^	92 (34.8)	27.92 ± 6.28		
Fixed night-shift system satisfaction		6.61 ± 2.08		-.24^†^	< .001
	0–3^a^	23 (8.7)	36.04 ± 5.33	18.33	< .001 a > b,c^‡^
	4–6^b^	77 (29.2)	32.04 ± 6.13		
	7–10^c^	164 (62.1)	30.23 ± 6.86		

Z = Mann-Whitney U test; H = Kruskal-Wallis test. ICU = Intensive care unit; M = Mean; SD = Standard deviation.

^†^Rho = Spearman’s rank correlation coefficients; ^‡^Post hoc pairwise comparisons were performed using Bonferroni correction.
